# Industrial Lead Poisoning

**Published:** 1900-07-28

**Authors:** 


### INDUSTRIAL LEAD POISONING.

In his annual report, Dr. T. M. Legge, the medical inspector of factories and workshops, gives a useful *résumé* of the kind of industries which have been productive of lead poisoning, as indicated by the notified cases. The list, however, is not to be regarded as complete, for there can be but little doubt that the duty which lies upon medical men to notify such cases is still unknown to many practitioners. A medical man, he says, is well aware of his duty to notify scarlet fever, &c., because it is an incident of his daily occupation. On the other hand, the majority of medical practitioners do not see a case of industrial lead poisoning from one year's end to another, and its notification is apt to be overlooked. Dr. Legge also alludes to the difficulty which sometimes surrounds the subject of notification in consequence of lead poisoning not presenting the same unmistakable signs that some acute infectious illnesses do, the large number of cases which on investigation prove to be doubtful—fully 10 per cent.—showing what difficulties medical men sometimes encounter in complying with the law. The fact is that "no absolute definition of lead poisoning is possible," and that "each practitioner in reporting what he believes lead poisoning to be must form his own standard, and has further to consider whether he should report subsequent attacks in the same person." Among the occupations of those notified as suffering from industrial lead poisoning were the smelting of metals, including other ores besides those nominally of lead; brass works; file cutting, an industry which is productive of a large proportion of severe cases, the number of cases of paralysis being greatly in excess of those in any other industry; printing, including the use of the linotype; tinning and enamelling of iron hollow ware; whitelead making, a work in which a considerable number of Italians have lately been employed in the East of London—at first many of these people took the rules and regulations very easily, and were very careless about themselves, but the occurrence of a number of cases of plumbism, together with the printing of the rules and regulations in Italian, has drawn their attention to the matter, and they are now said to be more careful; red lead making; china and earthenware work; glass polishing; enamelling of iron plates; making electrical accumulators; making paints and colours; coach painting, the work of sand-papering the paint to make the fresh coats adhere, and that of burning off the old coats of paint being especially alluded to as productive of the disease; shipbuilding, from the use of red and white lead in making joints; and, finally, house-painting, in regard to the notification of which there is considerable difficulty, for, so far as work is not carried on in a workshop or a factory, the Home Office has no power to interfere, and, indeed, has no power to pay for notification. If, however, a house painter spends some portion of his time in a factory or workshop, in processes such as grinding lead pigments or mixing lead paint, "the question may legitimately arise whether his case does not become reportable, seeing that the risk, though in a minor degree, is incurred there as well as in his usual outdoor work." The practice has been to include in the return under "other industries" such cases in house painters in which this state of things existed, and to regard the others as non-notifiable. This has led in some cases to slight friction with the medical practitioners notifying, who are inclined to regard any distinction in industrial lead poisoning as pedantic. The practice now adopted, then, is to accept the notification on the assumption (1) that the practitioner is in a position to say that the patient is employed in a given factory or workshop in which from the nature of the work carried on risk of lead poisoning may reasonably be anticipated; and (2) that no case will be notified in which this is not so. This is a comfortable way out of the difficulty, no doubt, at the same time we cannot but feel that it would be far better if the whole system of notification of disease were simplified, all notifications which are required by law being sent to one authority—the sanitary—and then communicated as a mere office detail to the other bodies involved, be they factory inspectors, school boards, or what not. It is enough for a doctor to have to certify to what he knows—namely, that his patient is suffering from lead; it is mere playing with certificates to ask him to certify that the patient got the poison in the course of his work; and still more to divide a man's work into two portions, one certifiable and the other non-certifiable, yet both involving the use of lead, and then to ask the doctor to decide in which part of his work he became affected. In any case, the notification of industrial poisoning alone is a very one sided business. If notification is to be made compulsory at all it should apply all round, independently of causation, and certainly the notification should in all cases alike be made to the medical officer of health rather than to the factory inspector. The law, however, says otherwise, and we must do as we are told; but it is to be hoped that some time things will be placed on a more logical basis.

